# Understanding How Minerals Contribute to Optimal Immune Function

**DOI:** 10.1155/2023/3355733

**Published:** 2023-11-01

**Authors:** Alina Stefanache, Ionut-Iulian Lungu, Ioan-Adrian Butnariu, Gabriela Calin, Cristian Gutu, Constantin Marcu, Carmen Grierosu, Elena Roxana Bogdan Goroftei, Letitia-Doina Duceac, Marius Gabriel Dabija, Florina Popa, Daniela Damir

**Affiliations:** ^1^“Grigore T. Popa” University of Medicine and Pharmacy, Iasi 700115, Romania; ^2^Faculty of Dental Medicine, “Apollonia” University of Iasi, 11 Pacurari Street, Iasi 700511, Romania; ^3^Faculty of Medicine and Pharmacy, University Dunarea de Jos, 47 Domneasca Street, Galati 800008, Romania

## Abstract

Sufficient mineral supply is vital not only for the innate immune system but also for the components of the adaptive immune defense, which encompass defense mechanisms against pathogens and the delicate balance of pro- and anti-inflammatory regulation in the long term. Generally, a well-balanced diet is capable of providing the necessary minerals to support the immune system. Nevertheless, specific vulnerable populations should be cautious about obtaining adequate amounts of minerals such as magnesium, zinc, copper, iron, and selenium. Inadequate levels of these minerals can temporarily impair immune competence and disrupt the long-term regulation of systemic inflammation. Therefore, comprehending the mechanisms and sources of these minerals is crucial. In exceptional circumstances, mineral deficiencies may necessitate supplementation; however, excessive intake of supplements can have adverse effects on the immune system and should be avoided. Consequently, any supplementation should be approved by medical professionals and administered in recommended doses. This review emphasizes the crucial significance of minerals in promoting optimal functioning of the immune system. It investigates the indispensable minerals required for immune system function and the regulation of inflammation. Moreover, it delves into the significance of maintaining an optimized intake of minerals from a nutritional standpoint.

## 1. Introduction

Minerals are indispensable components of our dietary intake, performing a diverse array of functions. They serve as the fundamental elements for our skeletal structure, influence muscle and nerve activity, and regulate the body's hydration balance. Furthermore, they are integral parts of hormones, enzymes, and other biologically active substances [1]. Several minerals play a critical role in optimizing the functionality of the immune system. Both the innate immune system and the adaptive immune response are influenced by their presence. Consequently, the availability of minerals can impact susceptibility to infections as well as the manifestation of chronic diseases [2, 3]. For most individuals, maintaining a well-balanced diet is sufficient to provide the body with the essential minerals at necessary levels. However, an increasing number of people are currently susceptible to mineral deficiencies. These include older individuals, those with chronic health conditions, vegetarians and vegans, as well as pregnant women. Athletes who adhere to restrictive or unbalanced diets, such as for weight loss purposes, may also be at risk of experiencing mineral deficiencies. In such cases, it is advisable to consult a qualified nutritionist to determine the appropriate composition and dosage of specific minerals, taking special consideration of any potential drug interactions [4].

If individuals possess sufficient knowledge about minerals, their significance, and the food sources that contain them, they can easily adjust their diets to compensate for any potential mineral deficiencies. The aim of this article was to delineate the immunological roles of specific minerals and provide recommendations for a well-balanced diet that incorporates these minerals. With the available research, we focused on minerals that exhibit a robust correlation with immune function.

## 2. Materials and Methods

### 2.1. Searched Databases

The searched databases were as follows: PubMed, SciELO, Google Scholar, and sites of known international organizations, such as the World Health Organization and the International Pharmaceutical Federation.

### 2.2. Keywords

All keywords were conveniently selected based on their connection with the present topic. The searched strings of keywords were as follows: magnesium, zinc, copper, iron, selenium and innate immune system, adaptive immunity, and infectious diseases.

### 2.3. Search Methodology and Data Extraction

The strings of keywords were introduced in the databases or websites. The same researcher conducted the search, selected the papers and other relevant documents/guidelines, and summarized the key findings. Papers (and other documents) were conveniently selected based on their pertinence and contribution to the present topic. Strengths, weaknesses, opportunities, and/or threats were identified and extracted by one author. Results were collected/organized in a tabular format (a Word document).

**Figure 1:**
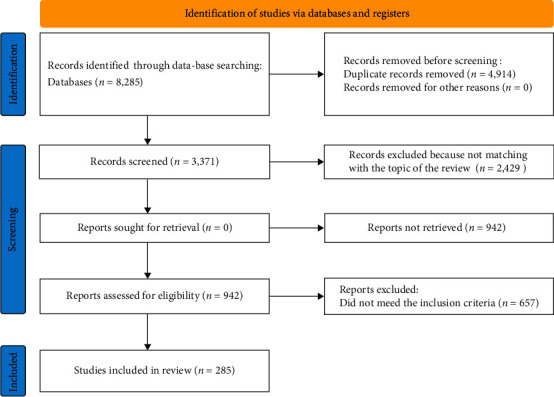
Identification of studies via databases and registers.

All study findings were double-checked for accuracy at the end. For instance, data were checked in the steps of (1) data collection and (2) elaboration/drafting of discussion. The extraction of data followed a saturation methodology, that is, the search was previously defined as concluded when the sequential analysis of five new works/papers did not result in any new output. All searches and data collection were manually carried out [5] (Figure 1).

### 2.4. Inclusion and Exclusion Criteria

Inclusion criteria were as follows: peer-reviewed papers or other documents (e.g., guidelines) describing the impact of minerals on immunity function. All types of papers were classified as eligible to be included, such as reviews, original research, and commentaries. Exclusion criteria were as follows: other topics or papers out of the covered timeframe.

### 2.5. Thematic Analysis

All identified topics were classified into groups/subcategories. These groups/subcategories were conveniently created based on a content analysis of the identified topics (i.e., general physiological functions, immunological roles, infectious diseases, and nutritional aspects for magnesium, zinc, copper, iron, and selenium).

### 2.6. Quality Assessment of the Present Narrative Review and the Selected Papers/Works

The quality evaluation of the present narrative review was carried out according to SANRA—a scale for the quality assessment of narrative review articles. Only peer-reviewed papers were selected, which constituted the only quality indicator. The works/documents from known international organizations were previously assumed to be acceptable since these documents are usually developed by recognized international experts.

## 3. Results

### 3.1. Magnesium (Mg)

#### 3.1.1. General Physiological Function of Magnesium

Magnesium primarily exerts its functions by binding to organic compounds, including proteins, nucleic acids, and nucleotides. It is the abundant divalent cation in living cells and serves several important roles in regulating cellular processes [6] as shown in Table 1.

Significant quantities of magnesium are present in the cells and matrix of bone. Moreover, higher concentrations can be observed in erythrocytes, blood serum, soft tissues, muscle cells, and blood. There is minimal disparity in magnesium concentrations between the intracellular and extracellular spaces. However, the potential at the cell membrane is influenced by the presence of free magnesium ions. Intracellular regulation of magnesium primarily occurs through an active transport mechanism [7, 10].

#### 3.1.2. Nutritional Aspects of Magnesium

The recommended daily intake of magnesium varies depending on factors such as age and gender. Different guidelines suggest different amounts, but typically the recommended dose ranges from 300 to 400 mg for males and 270 to 310 mg for females. It is worth noting that these recommendations may be slightly higher for older individuals. In fact, some researchers suggest an intake of up to 500 mg/day. It is important to consult the specific guidelines and recommendations provided by reputable sources and healthcare professionals to determine the appropriate magnesium dosage for your individual needs. Factors such as overall health, existing medical conditions, and medication interactions should also be taken into consideration when determining the optimal magnesium intake [11]. Fruits and vegetables, as well as nuts, seeds, and whole grain products, are the main natural sources of magnesium. Due to its high solubility, magnesium might be lost during cooking. Only 30%–40% of the magnesium that is consumed is thought to be absorbed through the digestive tract [3].

#### 3.1.3. Immunological Role of Magnesium

Mg has a variety of roles in the control of immunological processes, especially in relation to the operation of various immune cell subpopulations [12]. The innate immune system, adaptive immune system, and modulation of acute and chronic inflammatory processes are three examples of the many effects that can be used to highlight them [13].

*(1) Magnesium and the Innate Immune System*. Mg has an impact on the acute phase response and how macrophages function, such as how they react to cytokines. It has been demonstrated that magnesium supplementation decreases cytokine production following toll-like receptor (TLR) stimulation in monocytes. This immunoregulatory effect was brought about by a rise in IkB levels, which inhibited the translocation of the nuclear factor kappa-light chain enhancer of activated B cells (NF-B) [14]. Peripheral neutrophilia has been observed in Mg-deficient rats and is linked to an increase in phagocytosis and an oxidative burst [15].

*(2) Magnesium and Adaptive Immunity*. Lymphocyte growth, differentiation, and proliferation are significantly influenced by magnesium [16]. Apoptosis is hypothesized to be hampered in this situation by a magnesium deficiency. The process of Mg dependence in Fas-induced apoptosis is well recognized. The T-cell pool was negatively impacted by the thymus' early involution in mice with a magnesium deficiency [17]. Apparently playing a unique role in the maturation of T cells is the Mg^2+^ transporter TRPM7. Early cell death and developmental inhibition occurred in cells lacking this transporter and, consequently, the Mg supply [18]. Accordingly, both at the level of the individual cell and with reference to an individual's T-cell pool, T-cell function appears to be dependent on an adequate supply of magnesium. The regulation of phosphoinositide metabolism by Mg^2+^ from intra- and extracellular sources has also been demonstrated in a number of investigations in lymphocytes [19]. Because correct substrate metabolism is required for several immune cell functions, including proliferation, magnesium may indirectly alter these processes [20].

Magnesium is an essential cofactor for several phosphorylation-related enzymes, including those involved in the phosphorylation cascades of glycolysis and nucleotide polymerization [20]. Accordingly, it is hypothesized that the magnesium that is released during acute inflammation is beneficial for T cells' metabolic activity, which controls key processes like proliferation.

*(3) Immunoregulating Effects of Magnesium*. Insufficient magnesium levels seem to contribute to an overactive innate immune system and a weakened adaptive immune system. This observation potentially underlies the findings of certain human studies that suggest a connection between magnesium deficiency and chronic, low-grade inflammation. Magnesium plays a crucial role in modulating immune responses by influencing various immune cells, cytokines, and signaling pathways. It has been shown to regulate the activation and function of immune cells, including macrophages, T cells, and B cells. Moreover, magnesium is involved in the production and release of inflammatory mediators, such as cytokines and chemokines. The deficiency of this essential mineral may disrupt the delicate balance of immune regulation, leading to chronic inflammation and altered immune responses [21]. This has been demonstrated, for instance, by an inverse correlation between low serum Mg concentrations and elevated systemic C-reactive protein (CRP) levels [22]. Although there is evidence from in vitro research demonstrating that magnesium shortage results in increased production of interleukin (IL) and tumor necrosis factor (TNF), the causal relationship is not totally clear. Increased platelet aggregation brought about by a magnesium deficit also had an effect on how well the microvasculature worked. According to these results, a recent meta-analysis demonstrated that magnesium supplementation lowers serum CRP levels [23]. These investigations, however, involved a small number of participants, indicating the need for additional study.

Experimental mice subjected to a magnesium-reduced diet exhibited a sudden and severe magnesium deficit, resulting in several indications of an altered proinflammatory state. The levels of systemic IL-6, a proinflammatory cytokine, increased, along with the production of acute phase proteins. Concurrently, oxidative stress markers, such as thiobarbituric acid-reactive compounds, showed an increase. The activity of superoxide dismutase (SOD) and catalase, enzymes involved in antioxidant defense, both decreased. Furthermore, there was a reduction in the magnesium-dependent process of glutathione synthesis, a key antioxidant mechanism. It is likely that the release of Substance P, a proinflammatory neuropeptide associated with magnesium deficiency, contributed to the elevated oxidative stress. These findings suggest that magnesium deficiency can induce a proinflammatory state characterized by increased oxidative stress and altered antioxidant activity [24]. Even a temporary Mg deficit led to an increase in the production of ceramides, which are known to activate NFkB and, as a result, stimulate the release of a number of proinflammatory cytokines such as TNF-, IL-1, and IL-6. The proinflammatory effects of experimental hypomagnesemia were amplified by the disruption of Ca^2+^ homeostasis [25]. Evidence suggests that systemic magnesium deficiency also has an impact on the microbiome. In a state of magnesium shortage, the integrity of the intestinal barrier function is compromised, and there is a reduction in the concentration of bifidobacteria. This alteration in the microbiome composition contributes to increased expression of proinflammatory cytokines such as TNF and IL-6 (interleukin-6) in both the intestine and liver. The disruption of the intestinal barrier function can lead to increased permeability, allowing the translocation of harmful substances and bacterial components into the bloodstream, triggering an immune response and promoting inflammation. The decrease in bifidobacteria, which are beneficial bacteria with various immunomodulatory effects, further exacerbates the proinflammatory state. Therefore, magnesium deficiency not only affects the immune system but also has a significant influence on the gut microbiome and its associated inflammatory responses [26].

#### 3.1.4. Magnesium and Infectious Diseases

The significance of magnesium in the setting of infections is mostly attributed to the intimate relationship between the metabolism of vitamin D and the significance of magnesium as a cofactor. This suggests that less vitamin D can be synthesized from its precursors if magnesium is scarce [27, 28]. However, animal studies provide the majority of information on immunodepression and its relationship to magnesium status. These have verified that a lack of magnesium causes a variety of inflammatory response alterations that can affect the risk of infection [29].

### 3.2. Zinc

#### 3.2.1. General Physiological Function of Zinc

Zinc (Zn), an indispensable trace element, has been extensively studied in the field of nutrition and health due to its crucial role in numerous physiological functions in the human body, as shown in Table 2. Its essentiality and diverse range of functions make zinc a topic of great interest and importance in the field of nutrition and human health research [30].

An adult has a total Zn content of about 2–3 g, of which 85% is dispersed throughout the muscles and bones. The majority of the substance is transported through the bloodstream and stored in red blood cells by SOD and carbonic anhydrase [37]. Zn is, however, 60% bound to albumin in plasma, which has a total concentration of 12–16 *μ*M [38, 39]. Proliferation, differentiation, and apoptosis are all tightly correlated with the equilibrium of the cellular Zn status [40]. As a result, a lack of this element can contribute to immune system dysfunction, which can have a serious effect on one's health.

#### 3.2.2. Immunological Role of Zinc

Due to its crucial role in various physiological functions, zinc deficiency can have a significant impact on the immune system, which exhibits a high rate of cell division. The immune system relies on zinc for proper functioning, including the development and maturation of immune cells, the production of antibodies, and the regulation of immune responses. Zinc deficiency can impair immune cell proliferation and differentiation, compromise the production and activity of immune cells, and disrupt the balance between proinflammatory and anti-inflammatory responses. Consequently, individuals with zinc deficiency may experience increased susceptibility to infections, delayed wound healing, and impaired immune responses. Ensuring an adequate intake of zinc is vital for supporting optimal immune system function and maintaining overall health. Dietary sources rich in zinc include seafood, meat, legumes, nuts, and seeds [41]. The innate immune system and the adaptive immune system are the two main immune response subsystems [42]. The innate immune system's cells, particularly polymorphonuclear cells (PMNs), macrophages, and natural killer (NK) cells, are the first cells to function in pathogen identification and elimination when pathogens enter the body. The very first cells to actively infiltrate the infection site are PMNs. Through the process of phagocytosis, they capture pathogens and kill them by releasing reactive oxygen species (ROS) [35]. T and B lymphocytes, two highly specialized cells, are in charge of directing the adaptive immune response. T lymphocytes are involved in cell-mediated immune responses by activating other immune cells (T helper lymphocytes) and by producing toxic granules in cytotoxic T lymphocytes, whereas B lymphocytes are involved in the humoral immune response through the production of antibodies specifically targeted against an antigen. The innate and adaptive immune systems communicate with one another via dendritic cells (DCs). DCs circulate as immature cells, and when they come into contact with the antigen, they begin to express MHC molecules and coreceptors for the activation and stimulation of T cells.

*(1) Zinc and the Innate Immune System*. Proper functioning of both the innate and adaptive immune systems is essential for the optimal absorption and utilization of zinc. The innate immune system acts as the first line of defense against pathogens, providing immediate, nonspecific immune responses. Zinc plays a crucial role in supporting the functions of innate immune cells, such as neutrophils, macrophages, and NK cells, which are involved in phagocytosis, inflammation, and the elimination of pathogens. On the other hand, the adaptive immune system mounts specific immune responses by recognizing and targeting specific pathogens. Zinc is involved in the development and function of various adaptive immune cells, including T cells and B cells. It is required for T cell activation, proliferation, and differentiation, as well as for the production of antibodies by B cells. In addition to its direct effects on immune cell function, zinc is also involved in the regulation of cytokines, which are signaling molecules that coordinate immune responses. It helps maintain the balance between proinflammatory and anti-inflammatory cytokines, ensuring an appropriate immune response. Furthermore, zinc deficiency can impair the integrity and function of the gastrointestinal tract, which is critical for the absorption of nutrients, including zinc itself. This can create a vicious cycle, as zinc deficiency can further compromise immune function and lead to increased susceptibility to infections.

Overall, the proper functioning of both the innate and adaptive immune systems is essential for the optimal absorption and utilization of zinc, while zinc, in turn, plays a crucial role in supporting immune cell function and immune system regulation [43]. Zn is essential for the nicotinamide adenine dinucleotide phosphate oxidase activity of neutrophil granulocytes in the setting of the innate immunological response [44, 45]. Therefore, a Zn deficit may result in decreased production of ROS with a decreased capacity to kill [43]. In addition, in vivo research has demonstrated that Zn shortage impairs macrophage maturation and activity and reduces the adhesion and chemotaxis of monocytes and neutrophil granulocytes [46]. Zn has a crucial role in NK cells as well. Zn deficiency can result in a lower number of NK cells in the peripheral circulation and affect how well they function. In this respect, it has been shown that tumor cells or cells infected with viruses exhibit impaired chemotaxis and lysis [47, 48].

Through mechanisms that are still being fully elucidated, Zn controls a number of key procedures associated with the innate immune response. First, some immune cells are chemoattracted by Zn ions. Inversely, a superphysiological Zn concentration (500 *μ*M) increases PMN chemotaxis in vitro [49], and a Zn shortage reduces PMN chemotaxis [50]. Phagocytosis is reduced by Zn deficiency, but Zn supplementation has the opposite effect [51]. The early endosome antigen 1 (EEA1) and other Zn proteins involved in phagocytosis are likely how Zn affects this process. In order to facilitate membrane tethering and fusion, which are essential for phagosome and endosome maturation, EEA1 binds directly to the phospholipid phosphatidylinositol 3-phosphate (PI3K) at its C-terminal and binds to Rab5 via its *N*-terminal zinc finger domain [52, 53]. Zn is equally crucial for the neutralization of pathogens since both Zn excess and deficiency inhibit NAPDH, which controls the formation of superoxide anion that causes pathogen death following phagocytosis [54].

Zinc is not only essential for the proper functioning of the immune system but also plays a crucial role in the generation of proinflammatory cytokines. Cytokines, such as interleukins IL-1*β*, IL-6, and tumor necrosis factor *α* (TNF-*α*), are key mediators of immune responses. Zinc is required for the production and release of these proinflammatory cytokines, which are involved in initiating and coordinating immune reactions against pathogens. Moreover, studies have demonstrated that zinc promotes the adherence of monocytes, a type of immune cell, to endothelial cells in laboratory settings. This interaction is significant as it facilitates the migration of monocytes to sites of infection or inflammation, allowing them to exert their protective functions. In addition to its role in immune response modulation, zinc has been shown to possess antimicrobial properties. It can directly inhibit the replication and proliferation of various pathogens, including viruses and bacteria, thus contributing to the defense against infections. It is important to note that maintaining an appropriate balance of zinc is crucial, as both zinc deficiency and excess can negatively impact immune function. While insufficient zinc levels can impair immune responses, excessive zinc can lead to immunosuppression and disrupt immune cell signaling [55]. Zn deficiency only impacts the generation of IL-6 and TNF-*α* in an in vitro culture of peripheral blood mononuclear cells, leaving out the phagocytosis and oxidative burst of monocytes. This shows that under Zn deficiency, basic innate immune processes take the place of intracellular communication [56]. In addition, Zn modifies the response of NK cells by enhancing their MHC-class I expression and decreasing recognition [57]. In addition, Zn supplementation improves CD34+ cells' capacity to differentiate into NK cells and their cytotoxicity [58].

DCs' ability to mature is similarly impacted by Zn. Dynamic changes in the expression of Zn transporters on the cell surface are seen during LPS-induced DC maturation: ZIP6 and ZIP10 are inhibited while numerous ZnTs are up-regulated [59]. In addition, LPS stimulation increases the expression of MHC-II and costimulatory molecules on DCs while decreasing free Zn in DCs. The up-regulation of MHC-II and costimulatory molecules is interestingly inhibited by Zn supplementation or ZIP6 overexpression, while the LPS impact is interestingly mimicked by Zn chelator therapy [59]. These findings imply that DC maturation and subsequent activation of the adaptive immune response depend on a decrease in Zn in DCs through ZIP-6 down-regulation.

*(2) Zinc and Adaptive Immunity*. Zn has a significant role in the development, maturity, and functionality of T cells, which are crucial aspects of the adaptive immune system [32, 60]. This is due to the fact that Zn plays a crucial structural role in the hormone thymulin, which is created by the epithelial cells of the thymus and facilitates the maturation of pre-T lymphocytes into T lymphocytes [61, 62].

Zn deficiency has a particularly negative impact on T lymphocytes' ability to grow and function. Thymic atrophy and consequent T-cell lymphopenia are brought on by zinc deficiency. In mice, a shortage of Zn during T-cell maturation causes a 50% reduction in the number of “potential” pre-T cells compared to “effective” T cells, which is accompanied by an increase in pre-T cell death [63].

Zn also plays a crucial role in the mechanisms involved in T-cell development [64]. The number of CD4+ T cells decreased in studies that caused a Zn shortage, which led to an imbalance in the CD4+/CD8+ ratio [65]. Clinically, it is believed that a significantly decreased CD4+/CD8+ ratio, such as one below 1.5, is a sign of immunological dysfunction or one of its causes and is hence predictive of the prognosis of a number of disorders. There may also be an imbalance between Th-1 and Th-2 cells within CD4+ cells. The Th-1 cells in this instance are in the foreground and exhibit a more pronounced decline. Consequences are, for example, reduced Th-1-mediated cytokine production of TNF-*α*, IL-2, or IFN-*γ* [57, 65].

Th-17 cells that promote inflammation are also harmed by a zinc shortage. IL-6-induced STAT3 activation during chronic inflammation is a key regulator of Th-17 cell formation, and Zn inhibits Th-17 cell development by reducing this activation [66]. For instance, Zn therapy decreases Th-17 growth in the animal model of RA known as collagen-induced arthritis [66]. A mild nutritional Zn shortage raises the ratio of memory T cells to naive T cells and cytotoxic T cells to total T cells [57], which may result in immune system malfunction and autoimmune responses [67].

The binding of zinc to metallothioneins (MTs) plays a pivotal role in controlling various cytokine-driven activities in T cells. Transcription factors STAT1 and STAT3, crucial for T cell survival and development, are regulated by MTs. The interaction between Zn and MTs can affect the phosphorylation process of these transcription factors. Consequently, this modulation leads to an increase in the production of interleukin-10 (IL-10) by Tr1 cells, a subset of non-FoxP3 regulatory T (Treg) cells. IL-10 is a potent immunosuppressive cytokine known for its ability to downregulate inflammatory responses. By promoting the production of IL-10, Zn–MT binding can modulate immune activity and contribute to the regulation of autoimmune processes. IL-10 acts by suppressing the production of proinflammatory cytokines and inhibiting the activation of immune cells involved in autoimmune responses. Furthermore, IL-10 plays a crucial role in maintaining immune homeostasis by balancing the immune system's reactivity. It helps prevent excessive immune responses that can lead to tissue damage and autoimmunity. By influencing the generation of IL-10 through Zn–MT interactions, zinc can impact the delicate balance between immune activation and regulation. Understanding the intricate relationship between Zn–MT binding, cytokine-driven T-cell activities, and IL-10 production provides insights into the complex mechanisms underlying immune regulation and the potential therapeutic implications. Further research in this area is essential to unravel the precise mechanisms and explore the therapeutic possibilities of modulating Zn–MT interactions for immune-related disorders and autoimmune diseases [68].

Zn alterations have a smaller impact on B cell growth and function than they do on T cells. Nevertheless, Zn shortage results in a decrease in B cells, which affects the maturation of immature and premature B cells [69] and the production of antibodies [70]. Zn addition has been tested as a supplement for immunization in people who are deficient, with contentious results [71, 72].

*(3) Immunoregulating Effects of Zinc*. In addition to its specific effects on immunological functions, the overall regulation of the immune system is closely associated with zinc status. Numerous studies have demonstrated a correlation between Zn deficiency and dysregulation of immune responses, leading to increased oxidative stress and systemic inflammation.

Zinc is involved in the regulation of various immune processes, including the activation and differentiation of immune cells, the production of cytokines, and the maintenance of immune cell functions. Insufficient levels of Zn can impair these essential immune functions, compromising the overall immune system's ability to mount an effective defense against pathogens, as shown in Table 3.

Overall, maintaining adequate Zn levels is crucial for the proper regulation of the immune system. It not only supports specific immunological functions but also helps prevent oxidative stress, control inflammation, and ensure the optimal activation of the adaptive immune response. Understanding the relationship between Zn status and immune regulation is vital for developing strategies to enhance immune health and prevent immune-related disorders associated with Zn deficiency [73–75]. Numerous inflammatory cytokines can be affected by zinc in terms of their signaling as well as their synthesis. A Zn shortage can also have a deleterious impact on the disease processes that are linked to chronic inflammation. Patients with systemic inflammatory disorders, such as rheumatoid arthritis, and a concurrent Zn shortage have been demonstrated to have higher levels of IL-1*α*, IL-1*β*, and IL-6 expression compared to those who consume more Zn [76, 77]. In addition, the chromatin architecture of the IL-1*β* and TNF-*α* promoters, which allow for the production of both genes, appears to be altered by long-term Zn deficiency [40]. Thus, Zn can be considered a trace element that inhibits the synthesis of proinflammatory cytokines and has a favorable impact on disease processes. Since T lymphocytes and macrophages are the primary producers of cytokines, the mechanisms indicated above may be the cause of this. The decrease in ROS also has a significant impact [78]. In addition, the evidence points to Zn's function as a contra-regulator of the NF-*κ*B signaling pathway. The pathway controls the expression of genes involved in apoptosis, innate and adaptive immune responses, and inflammatory processes, which in turn affect the expression of proinflammatory cytokines like TNF-*α*, IL-1*β*, or IL-6 [79]. Based on how Zn impacts the expression of the protein A20, one of the most significant inhibitory mechanisms exists. A20 is a Zn finger protein that is well known for being anti-inflammatory and for negatively regulating the NF-*κ*B signaling pathways initiated by the tumor necrosis factor receptor and TLR [73].

In addition, in vivo studies have demonstrated that Zn addition generates and stabilizes regulatory T cells (Treg) [80, 81]. Thus, Zn has a broad impact while still being unique to each type of cell.

#### 3.2.3. Zinc and Infectious Diseases

Zinc plays a significant role in the context of viral infections, and emerging studies have consistently shown its positive impact on disease progression, and in some cases, its potential to prevent infections altogether. The mechanisms by which Zn exerts its antiviral effects involve various stages of the viral lifecycle, including viral particle entry, fusion, replication, translation of viral proteins, and release, encompassing a wide range of viruses. One crucial aspect of Zn's antiviral activity is its ability to inhibit viral entry into host cells. Zn can interfere with the attachment and internalization of viral particles, thereby preventing the initial steps of infection. By blocking viral entry, Zn reduces the viral load and limits the spread of the infection within the body. Moreover, Zn has been shown to inhibit viral fusion, which is the process by which the viral envelope fuses with the host cell membrane, allowing the release of viral genetic material into the cell. By disrupting this fusion process, Zn impedes viral replication and further propagation.

Zn also plays a role in regulating viral replication within infected cells. It can interfere with the translation of viral proteins, which are essential for the production of new viral particles. By inhibiting viral protein synthesis, Zn limits the replication capacity of the virus and hampers its ability to spread. In addition, Zn has been found to enhance the antiviral immune response. It can boost the activity of immune cells, such as NK cells and T cells, which play a crucial role in recognizing and eliminating infected cells. Zn supplementation has been shown to enhance the cytotoxic activity of these immune cells, enhancing the body's defense against viral infections. The specific mechanisms by which Zn exerts its antiviral effects may vary among different viruses. However, the cumulative evidence suggests that maintaining optimal Zn levels is crucial for mounting an effective antiviral defense. Zn supplementation has shown promise in mitigating the severity of viral illnesses and preventing their progression. Further research is ongoing to explore the detailed molecular mechanisms underlying Zn's antiviral properties and to identify specific targets within the viral lifecycle. Understanding the interplay between Zn and viral infections can provide valuable insights for the development of novel antiviral strategies and interventions [82, 83]. Regarding clinical effectiveness, a meta-analysis's findings revealed that Zn supplementation at a level >75 mg/day considerably shortened the length of colds [84]. The elderly are a demographic that is particularly at risk in this situation. Increased susceptibility to infections and their severity are associated with immune system aging. In a sample of 55- to 87-year-old people, it was demonstrated that after 12 months of Zn supplementation (45 mg elemental Zn-gluconate/day), the incidence of infections was much lower. This was accompanied by a rise in plasma Zn concentration and a decrease in the production of TNF-*α* and indicators of oxidative stress [85]. The fact that Zn cations, in particular, have been demonstrated to inhibit SARS coronavirus RNA polymerase (RNA-dependent RNA polymerase) by lowering viral replication in in vitro studies is more support [86]. These important findings show that Zn can be considered as an active agent in the treatment of COVID-19 [87].

These studies highlight the importance of zinc in maintaining immunological health. However, other research indicates that excessive zinc intake, ranging from 100 to 300 mg/day, can have detrimental effects on immune function and potentially contribute to various health problems. In line with this, Deuster recommends a maximum daily zinc intake of 40 mg as a tolerable limit. These findings underscore the challenges faced in conducting research in this field, as it can be complex to determine the optimal dosage and balance of zinc for overall health and immune function [88].

#### 3.2.4. Nutritional Aspects of Zinc

Zn is distributed unevenly throughout the body, so an organism must consume it every day because it has no obvious storage space. 17% of the world's population, according to estimates, is at risk of not getting enough zinc [89]. 15% of Americans are thought to consume insufficient amounts of zinc. This estimate increases to 35%–45% for older adults [90–92]. Older adults have lower Zn absorption rates in addition to having inadequate dietary Zn intake [93]. Thymic atrophy, lymphopenia, decreased adaptive immunity, and increased susceptibility to infection are all symptoms of Zn insufficiency that are very similar to age-related immunological dysfunction [60, 94]. According to age, sex, and, for adults, phytate intake, there are different reference levels for Zn intake. These suggested values change from one nation to the next. For adult men and women, the US Food and Nutrition Board advises an intake of 11 and 8 mg/day, respectively [95]. Infants between the ages of 0 and 4 months should consume 1.5 mg of zinc daily, according to the German Nutrition Society. Teenagers between the ages of 15 and 19 should consume 11 mg of zinc/day for females and 14 mg of zinc/day for males. For females aged 19 and older with low, medium, and high phytate diets, respectively, the recommended intakes of zinc are 7, 8, and 10 mg/day; for males, the values are 11, 14, and 16 mg/day [96]. Zn-rich foods include milk, cheese, eggs, beef, and pork. Nuts, such as cashew and pecan nuts, as well as wheat or rye sprouts, are sources of vegetable Zn. Additional food sources of zinc are listed in Table 4. Despite encouraging results regarding the impact on immunological homeostasis, Zn supplementation should always be individualized for each person because too much Zn can have the opposite effect.

### 3.3. Copper

#### 3.3.1. General Physiological Function of Copper

The regulation of copper (Cu) levels in the body is crucial due to its involvement in various physiological processes. While copper is necessary in moderate amounts for proper physiological functioning, excessive levels of copper can have severe adverse effects. One essential role of copper is its participation as a cofactor in the respiratory chain, where it facilitates the transfer of electrons to oxygen. This process is vital for cellular respiration and energy production. Furthermore, copper plays a critical role in maintaining a healthy oxidative balance within the body. However, it is important to maintain the delicate balance of copper levels, as excessive copper can lead to detrimental health outcomes [98]. Normally, the human body only has 80–150 mg of copper. The trace element can also be found in bones and muscles; however, it is primarily concentrated in the liver. When necessary, it leaves the depots and enters the circulation; any excess copper is then eliminated by the liver into the bile. The intestine excretes the majority, and the urine takes care of the remainder. According to age and sex, the range of normal copper levels in the blood is between 74 and 131 g/dL. When the levels in the 24-hr urine of adults are below 60 g, the urine value can also be used as a guide [99]. Cu also holds special significance among the serum's micronutrients and has been linked to systemic immunological activation [100].

#### 3.3.2. Immunological Role of Copper

While the precise mechanisms by which copper contributes to the development and functioning of the immune system remain unclear, its importance in these processes is well recognized. Copper is necessary for the normal development and optimal functioning of the immune system. Deficiencies in copper levels have been associated with reduced effectiveness of cellular and/or humoral immune system aspects. This deficiency can increase the risk of infections, as copper plays a role in immune response modulation. Further research is needed to fully elucidate the specific involvement of copper in immune system processes and its impact on infection susceptibility [101]. Naturally, newborns are particularly susceptible to the detrimental effects of low copper levels on immune system function. Infants with Menkes disease (MD), a condition characterized by severe copper deficiency, experience frequent, and severe infections. The compromised immune system in these individuals leaves them more vulnerable to microbial pathogens, leading to a higher incidence of infections. Neonates with MD require special attention and care to address their copper deficiency and mitigate the associated risks to their immune system [102, 103]. In addition, giving underweight, copper-deficient children a copper supplement improved their ability to phagocytize germs [104]. Another sign of a copper shortage in humans is neutropenia, and even mild copper deficiency may impair the function of various immune cells (such as macrophages and lymphocytes). For instance, after an in vitro immunological challenge, the proliferation of peripheral blood mononuclear cells isolated from adult males fed a low-copper diet (0.38 mg/day) for 42 days was inhibited [105]. In addition, individuals' plasma copper levels were low, and some cuproenzymes' activity was suppressed. The innate immune response to bacterial infection may also require copper [106]. Collectively, these studies demonstrate significant roles for copper in immune system function, but conclusive evidence is absent, in part because marginal or subclinical copper deficiency could not be identified in human intervention trial participants.

On the other hand, diminished humoral and cellular immunological function results from a Cu shortage. Cu-deficient mice had a smaller thymus and a larger spleen, according to research. Lower T cell counts and neutropenia are also present. B cell and NK cell function, as well as mitogen-induced T-cell proliferation, are all compromised [105, 107]. As a result, raising plasma Cu levels may improve both innate and adaptive immunity in people. Cu is referred to in this context as a trace element with antiviral properties that can treat and prevent COVID-19 [108]. The body can prevent the growth of pathogens by limiting their access to Cu, yet Cu is also a necessary nutrient for microbial pathogens. The human body's intake of Cu should be balanced from the standpoint of the immune system because too much can simultaneously have negative effects while too little is necessary for proper immunological function [109].

*(1) Copper and the Innate Immune System*. While the effects of copper deficiency on neutrophils and macrophages are well understood, there is limited knowledge regarding its impact on eosinophils and basophils. Due to their low abundance, studying these two cell types presents challenges in research. It is worth noting that mast cells originate from basophils within tissues. In the cremaster muscle of mice with copper deficiency, further investigation is required to fully comprehend the specific consequences on eosinophils, basophils, and the development of mast cells. Schuschke et al. [110] discovered an increase in the mast cell population, indicating that copper shortage might affect the maturation patterns of the leukocyte population or the distribution of blood cells into tissues.

In humans, neutropenia is a clinical indicator of copper insufficiency [111, 112]. The 1960s were when it was first noticed [113]. Humans with copper deficiency had more promyelocytes and fewer metamyelocytes and banded cells in their bone marrow aspirates. This was understood to be a granulocyte maturation arrest brought on by a copper deficit [114–116]. Inadequate secretion from the bone marrow, early death of progenitor cells, a shorter life expectancy of the circulating peripheral cells, and redistribution into tissues or organs are some other processes that could cause neutropenia [117]. Antineutrophil antibodies were found by Higuchi et al. [118] in the serum of people with copper deficiency, which may point to a mechanism of neutrophil depletion. In addition to having fewer circulating neutrophils, the copper shortage also affected how well those neutrophils functioned. A copper shortage was observed to cause decreased superoxide anion generation and decreased candidacidal activity without altering phagocytosis [119, 120]. In macrophages, comparable modifications were seen [121]. Neutrophil dysfunction was also impacted by a minor copper shortage. Neutrophils drawn from the peritoneal cavities of rats with borderline copper deficiency in the perinatal model described earlier produced 60% less superoxide anion than did control rats' neutrophils [122]. This demonstrates once again that immunity is compromised even when copper status indices are within the normal range. However, despite our best efforts, we have not been able to identify whether the general population is marginally deficient in copper, despite the potential importance of the influence of marginal copper status on human health. The ideal quantity of copper one should eat has not been determined, although a recent study utilizing low-copper diets in young males revealed that the requirement for copper fell somewhere in the range of 0.4–0.8 mg/day [123].

*(2) Immunoregulating Effects of Copper*. The balance of Cu in the body is also influenced by nutrition. Balance studies have shown that daily intakes below 0.8 mg/day result in a net Cu loss, but net increases are seen beyond 2.4 mg/day [99]. It is worth mentioning that there seems to be an inverse relationship between the intake of zinc and copper. When significant amounts of zinc are consumed, lower levels of copper enter the body through the colon. This effect is utilized in the treatment of Wilson's disease, a condition characterized by copper overload, to regulate copper levels [100]. Contrarily, Cu shortages are extremely rare, occurring, for instance, in severe intestinal disorders. However, this frequently results in severe deficiency symptoms, like anemia and bone deterioration [109]. Cu is not a standard parameter since a high blood level of Cu is not necessarily harmful. Elevated levels can be seen in a number of illnesses, including cancer, infections, and diabetes mellitus; however, they are believed to have little impact on the course of the illness or the effectiveness of treatment [99].

#### 3.3.3. Nutritional Aspects of Copper

The recommended daily allowance (RDA) for copper intake is 0.9 mg/day [124], although the typical American adult diet comprises 1.0–1.6 mg of copper/day. The most abundant dietary sources of copper include shellfish, wheat bran cereal, nuts, seeds, organ meats, whole grains, and chocolate. Estimating the precise amount of copper in food can be challenging, and food composition charts may not always be reliable. While plant-based foods may decrease copper absorption, following a vegan diet does not necessarily lead to an increased risk of copper deficiency. Dietary supplements can also provide copper, but it's important to note that many supplements contain cupric oxide, which has limited absorption [125]. The RDA for copper intake in the US has been criticized as being insufficient. For instance, the most likely cause of up to 40% of the US population's low serum copper levels, which reflect a functional copper deficit [126], was suggested by the most recent National Health and Nutrition Examination Survey. These and other more recent experimental assessments back up the recommendation for adults to consume 2.6 mg/day of copper [127, 128].

### 3.4. Iron

#### 3.4.1. General Physiological Function of Iron

Iron (Fe), a vital dietary mineral, plays a crucial role in the development and functioning of the immune system, as well as processes like erythropoiesis (red blood cell production) and cellular energy metabolism. However, iron deficiency, which leads to anemia, remains a significant concern, affecting approximately 25% of the global population. Certain populations, such as females (both pregnant and nonpregnant) and children, experience a substantially higher prevalence of iron deficiency, ranging from 30% to 47% [129].

#### 3.4.2. Immunological Role of Iron

*(1) Iron and the Innate Immune System*. The handling of iron by macrophages is influenced by immune activation. In turn, the effectiveness of macrophages' antimicrobial immune response is directly influenced by their iron levels. Many of these interactions are attributed to the effects of iron on the binding activity of transcription factors that promote inflammation. Iron regulates the activity of various transcription factors, including NF-B, NF-IL6, HIF-1, STAT1, and Nrf2, although not consistently. Iron also stimulates the production of reactive oxygen intermediates (ROIs), which subsequently enhances NF-B activity [130]. A variety of proinflammatory cytokines, chemokines, antimicrobial enzymes, peptides, and adhesion molecules are transactivated by NF-B, one of the key transcription factors in the beginning and intensification of the immune response [131]. Because labile iron may catalyze the Fenton reaction, which produces oxygen radicals, it has strong proinflammatory effects. However, because NF-B activation also transactivates the Fth and Ftl genes, it also aims to reduce iron-induced cytotoxicity [132]. TNF has the ability to trigger apoptosis, while TNF-induced ROI promotes Ft expression via NF-B, starting the iron storage and antiapoptotic pathways [133].

NF-IL6, HIF-1, and STAT1 are frequently suppressed by cellular iron excess, in stark contrast to NF-B [134–136]. Iron lowers NF-IL6 activation and STAT1 phosphorylation, which inhibit Nos2 transcription and NO generation in macrophages treated with IFN and LPS to imitate TH1 immunity [134]. Similar to this, iron chelation with DFO stimulates HIF-1, which increases the transactivation of the Nos2 gene [135]. Bmp6 increases Nos2 expression in macrophages, which is intriguing and suggests that it is a key coordinator of iron homeostasis and the immune response [137].

However, there is limited understanding of the interplay between the SMAD1/5/8 signaling cascades and their relationship with iron. By contrast, there is substantial crosstalk between the NF-B and HIF-1 pathways. HIF-1 has the ability to inhibit gene expression induced by NF-B, while NF-B enhances the transcription of HIF-1 [138, 139]. HIF-1 and NF-B are both activated during infections, and they work together to increase the expression of target genes that are shared and crucial for host defense [140]. How iron might impact the interaction between these two important pathways is not yet known. Since the NF-B component p65 uses pirin as an interaction partner and redox sensor, it appears that pirin is the basis for the specificity with which iron drives NF-B activity. When under oxidative stress, pirin preferentially takes on its ferric form, which binds to p65 and increases its DNA-binding activity [141]. It's interesting to note that iron is required for HIF-1's stability and that the von Hippel Lindau complex and prolyl hydroxylases are involved in iron-mediated regulation of HIF-1's degradation [142]. NF-B-dependent Ft transcription is induced by TLR4 signaling, and Ft then integrates iron, lowering the metal's availability in the cytoplasm. Since iron serves as a cofactor for prolyl hydroxylases that degrade HIF-1, the decrease in free intracellular iron in DC induced by LPS results in HIF-1 stability [143]. It is plausible to suppose that this route influences the course of leishmaniasis because HIF-1 is crucial for host defense against *Leishmania*; however, it is unclear how much HIF-1 may influence the immunological response of macrophages that contain parasites [144]. HIF-1, however, is crucial for limiting bacterial reproduction in macrophages infected with *Streptococcus pyogenes* or *Pseudomonas aeruginosa* because it promotes the development of TNF, Nos2, and other antibacterial effectors. *S. pyogenes* can infect mice's skin more easily when HIF-1 is missing from myeloid cells [145].

In the case of HFE-associated HH, an autosomal-recessive illness, many of the impacts that iron has on macrophage effector functions have been examined. Caucasians make up 5% of the heterozygous population for the causative HFE gene deficiency, which is most frequently the C282Y mutation, and 0.225% of the homozygous population [146]. Hfe's ability to interact with Tfr1 on the cell surface, wherein binding to Tfr1 decreases its affinity for holo-Tf, is its primary function, according to research [147]. As a result, when Hfe is altered, TBI is taken up by hepatocytes and other parenchymal cell types at a higher rate. As a result, there is an increase in parenchymal iron deposition, which may be made worse by the relative lack of hepcidin caused by improper detection of blood iron in the absence of Hfe. The iron deficiency of monocytes and macrophages in HFE-associated HH appears paradoxical. While hepcidin levels in HH may have decreased in part as a result of this observation [146], Hfe itself may have adverse effects on iron release from monocytes and macrophages [148]. Because macrophage iron overload suppresses Lcn2 transcription, Hfe-deficient macrophages export more iron and develop an iron-depleted phenotype, which makes it easier to produce Lcn2 [149]. Based on Hfe's requirement for cytokine translation, iron in general and Hfe in particular regulate immune function on yet another level. Because the ribosome needs sufficient amounts of iron to translate these particular mRNAs, it has been demonstrated that Hfe/macrophages produce less TNF and IL-6 proteins [150].

In addition, macrophages are both hepcidin's targets and producers, offering a putative autoregulation mechanism and a further connection between iron homeostasis and immunity. Proinflammatory signals like IL-6 or PAMP cause the synthesis of macrophage hepcidin, which appears to be resistant to iron status [151–154]. Hepcidin affects macrophages in a paracrine and autocrine manner after being secreted [155]. Hepcidin produced by macrophages promotes Fpn1 internalization, which results in mononuclear iron retention in the AI [156]. In addition, it has been demonstrated that the connection between hepcidin and Fpn1 inhibits the production of TNF and IL-6 through Janus kinase-2 and STAT3 signaling, which activate suppressor of cytokine signaling-3 [157]. Hepcidin has anti-inflammatory effects on macrophages by regulating these signaling pathways and raising cellular iron concentration, which may be important for the shutdown of the innate immune response [158]. This impact can be partially attributed to iron's direct inhibitory effects on immune effector pathways triggered by IFN and LPS, such as the production of TNF, IL-6, or Nos2 [136, 159–161].

Another transcription factor that links innate immunity and iron homeostasis is Nrf2. The role of Nrf2 in the cellular response to oxidative stress has long been understood. Therefore, Nrf2 binds to the promoter region of genes having an antioxidant response element (ARE) and is activated by stressors such as iron-induced ROI and NO [162–164]. It's significant that some iron-related genes also have AREs in their regulatory regions. In order to control oxidative stress and maintain iron homeostasis, Nrf2 stimulates the expression of Hmox1, Fth, and Fpn1 [165, 166]. As a result [167], Nrf2 serves as a molecular bridge between these two processes. In conclusion, macrophages control the system's iron homeostasis, and iron has an impact on various aspects of the effector activities of macrophages, including the transcriptional and translational control of substances that cause inflammation.

#### 3.4.3. Iron and Infectious Diseases

*(1) Extracellular Infections*. An important factor in determining the course of infectious disorders is the regulation of iron homeostasis on both the mammalian and microbial sides [168]. Both adversaries work to meet their iron needs when the host and pathogen interact. Because iron is necessary for almost all infections, its availability alters immune effector pathways as well as microbial proliferation [169–174]. The only bacteria that apparently do not require any iron at all but instead use other divalent metals are those belonging to the genus Lactobacillus, which are commensals, and *Borrelia burgdorferi*, which causes Lyme disease [175, 176]. Therefore, one effective mammalian defense approach to prevent the development and pathogenicity of almost all microorganisms is iron sequestration in areas that are inaccessible to invasive bacteria. It's important to note that the mechanisms of iron withdrawal differ fundamentally depending on whether infections are located extracellularly or intracellularly.

An excess of iron worsens the course of many infectious disorders and raises the risk of bacterial infections. Patients with genetic and acquired hemologic diseases who require frequent packed RBC transfusions have been shown to have a substantial positive correlation between infections and iron overload. For instance, blood transfusions, inefficient erythropoiesis, and increased iron absorption all contribute to iron overload in thalassemia syndromes [177, 178]. Although excess iron is hazardous to parenchymal organs like the heart and liver, it also increases the risk of infection. In fact, patients with *β*-thalassemia have been reported to have invasive infections with *Streptococcus pneumoniae*, *P. aeruginosa*, *Klebsiella* species, *Yersinia* species, *Escherichia coli*, and *Vibrio vulnificus*, and their risk is directly correlated with the degree of iron overload and a delay in therapeutic iron chelation [179]. The pathophysiology of inborn hemolytic anemias, such as thalassemias, is complex, and there may be a number of factors contributing to the higher incidence of infections, such as functional problems with the enlarged spleen and immunological dysregulation at many levels. In addition, hemolysis, tissue iron overload, and a rise in serum iron above Tf's binding capacity make it simple for bacteria to reach iron sources. The latter hypothesis is supported by the observation that *V. vulnificus* induces lethal septicemia in Hamp/Mice, a mouse model of extreme iron overload, showing that iron overload predisposes to a catastrophic outcome of normally uncommon infections [180]. Importantly, infections are a major contributing cause of iron overload's detrimental effects on leukemia and myelodysplastic syndrome patients' survival after stem cell transplantation [181, 182].

Numerous ways have been developed by microbes to exploit host iron supplies. Numerous extracellular bacteria contain Hb, heme, Ft, or Tf receptors. As a result, haptoglobin and Hpx, two acute phase proteins that bind free Hb and heme for later uptake into the MPS via CD163 and CD91, respectively, reduce the amount of iron that is available to bacteria [183, 184]. To reduce the pool of TBI during bacteremic episodes, Tf itself is downregulated during infection and is also eliminated from the blood via Tfr1. Although serum Ft is iron-poor in and of itself, it can also be removed from the bloodstream by Ft receptors on macrophages [185]. As a result, the Ft receptors on macrophages and microbes appear to be in direct competition for the same ligand that carries iron [186].

By employing iron-binding siderophores, which they either generate themselves or receive from other microbes but use as an iron supply, many bacteria, including mycobacteria and enterobacteriaceae like *E. coli* and *Klebsiella* species, kidnap iron from TBI [187–190]. Siderophores are various chemically structured, tiny molecular-weight iron chelators that share two characteristics. They exhibit an incredibly high affinity for ferric iron and are enzymatically produced outside of the ribosome [191–193]. In fact, siderophores can remove iron from host molecules like Tf which have a high yet low affinity for iron. Bacteria then absorb iron-rich siderophores through certain receptors found in their outer membrane. Certain fungi as well as bacteria can create siderophores. For example, *Aspergillus fumigatus* has both external siderophores that are associated with its pathogenicity and an internal siderophore for storing iron [194–196].

Given the significance of siderophore-mediated iron uptake for bacterial pathogenicity, mammals have a unique defense system called lipocalin-2 (Lcn2) that can reduce part of the threat provided by siderophores. Neutrophils, macrophages, and other immunological and parenchymal cell types all produce the peptide Lcn2, which has antibacterial and immune-regulating properties [197–204]. Its primary purpose is to bind siderophores of the catechol type, like enterobactin, to prevent iron from moving to bacteria [187, 205]. Due to the high susceptibility of Lcn2 mice to enterobacteriaceae like *E. coli*, this pathway appears to be particularly significant in these infections [206]. *Salmonella* species alter the structure of enterobactin to evade Lcn2's effects. Enterobactin is modified by enzymes and receptors produced by the iroA gene cluster to create salmochelins, which are too large to be neutralized by Lcn2 but still have the ability to bind iron [207]. Salmochelins do, however, need particular efflux pumps and membrane receptors [208, 209]. Lcn2 is particularly resistant to other siderophores made by *Pseudomonas pyoverdin*, *Yersiniabactin* generated by *Yersinia* and *Klebsiella* species, and triacetylfusarinine C produced by *A. fumigatus* [210–212]. In addition, Lcn2 guards against possible pathogens colonizing or infecting the gut mucosa [213, 214]. In addition, Lcn2 may be important for host defense against a wider variety of extracellular microorganisms because it attracts neutrophils to the site of infection [198]. Due to reduced iron availability in the macrophages' milieu, Hfe/macrophages and mice were protected from *Salmonella typhimurium* infection in a mouse model of HFE-associated HH by enhanced Lcn2 production [215]. However, when *Yersinia pestis*, the plague-causing extracellular bacterium, infected an individual with HFE-associated HH, the clinical course was unfavorable because elevated serum iron levels and parenchymal iron overload promote pathogen proliferation [216]. It is unknown how much the relative hepcidin shortage that characterizes HFE-associated HH may be responsible for this behavior. However, *Yersinia enterocolitica*, a closely related disease, is extremely virulent in Hamp/mice, indicating that Hfe and Hamp are crucial for host defense against *Yersinia* species [217, 218].

*(2) Intracellular Infections*. Intracellular infections are a major worry for world health. Serious infections can be brought on by *Mycobacterium* species, *Listeria monocytogenes*, *Legionella pneumophila*, *Chlamydia*, *Salmonella*, and *Leishmania* species. In addition, a variety of parasites, notably *Trypanosoma* and *Plasmodium*, have intricate, partially intracellular life cycles [219]. Some of these infections are becoming more resistant to antibiotics, and there aren't yet effective vaccinations available for all of them. Therefore, it's crucial to have a better understanding of immunity, particularly dietary immunological techniques like iron withdrawal, to increase the range of available treatments for illnesses like malaria, typhoid fever, and tuberculosis. One innovative method for treating infections caused by intracellular microorganisms is the pharmacological manipulation of iron homeostasis in host cells [220].

The iron exporter Fpn1 is a good candidate for an augmentation treatment in infections with intracellular pathogens. This theory is supported by the observation that Fpn1 regulates the reproduction of iron-dependent pathogens within host cells as well as macrophage effector functions [221]. For instance, overexpression of Fpn1 decreases the amount of iron available to intramacrophage bacteria and consequently the microbial burden in infections with *Chlamydia psittaci*, *L. pneumophila*, *Mycobacterium tuberculosis*, or *S. typhimurium* [222–224]. Exogenous hepcidin, on the other hand, promotes the growth of infected cells [222, 224, 225]. It is significant to note that several intracellular pathogens, including *Chlamydia pneumoniae*, *L. monocytogenes*, *S. typhimurium*, and *Trypanosoma brucei*, have been shown to induce intrinsic Fpn1 in infected macrophages [226–229]. This suggests that intracellular PRR senses microbial structures or metabolites and induces iron export to deprive the pathogenic invader of the micronutrient iron. Nos2's output, NO, promotes Nrf2 to transactivate Fpn1 transcription in *S. typhimurium*-infected macrophages, whereby Fpn1 is induced [230]. This does not rule out the possibility that different Fpn1 regulatory pathways could predominate in infections with other intracellular bacteria. For instance, *L. monocytogenes* replicates in the cytosol of the macrophage, while *S. typhimurium* infects murine macrophages via intraphagolysosomal infection. Therefore, it is plausible to believe that a separate molecular mechanism may be involved in the induction of Fpn1 transcription in response to *L. monocytogenes*. In addition, Fpn1 expression may be differentially regulated over the course of infection or between different organs that harbor pathogens, potentially allowing for systemic iron redistribution to maintain vital processes like erythropoiesis without interfering with nutritional immune strategies [231–234].

The phagolysosomal membrane of macrophages infected with *Mycobacterium* TB has also been shown to contain Fpn1. Its role there and how it interacts with Nramp1, also known as Slc11a1 (for natural resistance-associated macrophage protein-1), with which it colocalizes, are still unknown. Fpn1 may transfer iron from the cytoplasm into the phagosome, assuming normal orientation and potentially enhancing ROI formation. Accordingly, moderate dietary iron supplementation promotes ROI production, which lowers mycobacterial burden [235]. Contrarily, the bulk of papers on Nramp1's function contend that it serves as a mechanism for nutritional withdrawal by transporting iron out of the phagosome. Given that hepcidin was also discovered in the phagosomal area [236], the picture of Fpn1's probable function in the phagolysosome becomes even more complicated. However, it is still unclear whether it interacts with Fpn1 there or if its defensin-like structure enables it to perform direct antimicrobial actions.

In mice, Nramp1 was initially identified as a protein necessary for the management of intracellular pathogen infections, particularly those caused by *Leishmania* species, *Mycobacterium* species, and *S. typhimurium*. It was presumptively believed that Nramp1 must target a metabolic pathway important to both bacteria and protozoa because both pathogens are taxonomically unrelated. Because Nramp1 functions as an antiporter of protons and divalent metal ions, including iron, zinc, and manganese, across the phagolysosomal membrane, iron was found to be exactly at the center of that pathway [237, 238]. To specifically remove these ions as vital nutrients from absorbed iron-sensitive bacteria, Nramp1 transfers them from the phagolysosomal compartment into the cytoplasm. Nramp1 performs immune-regulatory tasks concurrently. A single nucleotide polymorphism (Nramp1s) within the coding region has been demonstrated to impair Nramp1's transport activity in mice. By contrast, the functional Nramp1r allele defends against infection with *S. typhimurium*, *Mycobacterium* species, and *Leishmania* species. Nramp1r macrophages produce more NO, TNF, IL-6, and Lcn2 and less of the anti-inflammatory cytokine IL-10 when *S. typhimurium* is present [239–242]. These findings support the hypothesis that Nramp1 promotes a proinflammatory macrophage phenotype, regulates the immune system, and helps in the eradication of intraphagolysosomal pathogens.

However, Nramp1's immune-regulatory effects seem to have a price. To date, no mutation in the NRAMP1 coding area in humans has been reported. Instead, polymorphisms in the NRAMP1 promoter's regulatory region control whether the gene is transcribed at higher or lower levels. An increased risk of autoimmune diseases such as rheumatoid arthritis, type 1 diabetes, and inflammatory bowel disease was linked to higher expression levels of NRAMP1. *Leishmania*, *Mycobacterium leprae*, and *Mycobacterium* TB infections, on the other hand, rose in frequency when promoter variations that induce low levels of NRAMP1 expression were present [243, 244]. It is tempting to hypothesize that these effects are related to different iron availability, which influences the balance of pro- and anti-inflammatory cytokines and, in turn, regulates tissue damage and the host's response to intracellular infections. In addition, Nramp1 is necessary for effective iron recycling after erythrophagocytosis, adding yet another crucial mechanism that may be able to manage both hemolysis and host defense simultaneously [245].

LIT1, a ferrous iron ZIP (for ZRT- and IRT-like protein) transporter discovered in *Leishmania* species, may directly compete for Nramp1's substrate in Leishmaniasis. When *Leishmania* is present in the phagolysosome, where it may obtain iron, LIT1 is specially produced. Accordingly, Nramp1r macrophages express more LIT1 [246]. In addition, as shown by the elevated IRP-binding activity of infected macrophages, *Leishmania* takes up hemoglobin and targets the labile iron pool that is present in the cytoplasm of macrophages [218, 247, 248].

#### 3.4.4. Nutritional Aspects of Iron

To ensure optimal human function, including oxygen transport and energy metabolism, as well as proper immune response against pathogens, maintaining a healthy iron (Fe) status appears to be beneficial. Moreover, the body's innate response to infection involves reducing the availability of extracellular iron, which limits the pathogen's ability to obtain nutrients from the host. It is worth noting that individuals with compromised health due to illness or infection may temporarily modify their behavior, such as reducing iron supplementation or consuming fewer iron-rich foods [170]. These changes may help the body's natural Fe withdrawal response.

### 3.5. Selenium

#### 3.5.1. General Physiological Function of Selenium

Selenium (Se) is an essential trace element that plays a critical role in maintaining homeostasis in both humans and animals. Approximately 50% of the body's selenium is found in skeletal muscle. Selenium is incorporated into the selenoprotein glutathione peroxidase (GPx), where it acts as an antioxidant [249]. Importantly, Rayman [250] notes that the importance of selenoproteins to health has been highlighted by the finding of disease-associated polymorphisms in selenoprotein genes. For instance, the thyroid gland's functions depend on selenoproteins, and GPx specifically safeguards the thyroid gland by removing too much hydrogen peroxide [251].  Table 5 lists further physiological actions of Se.

#### 3.5.2. Immunological Role of Selenium

The immune system must have enough selenium to function properly. Individual selenoproteins control inflammation and immunity, and Huang et al. [252] explore the ways in which Se affects these processes in depth. In addition, they discussed how Se levels affected autoimmunity, sepsis, allergic asthma, and chronic inflammatory diseases. It is crucial to remember that supplementation is likely to only help people whose intakes are insufficient.

*(1) Immunoregulating Effects of Selenium*. Selenium deficiencies are significant, although relatively uncommon. The degree of this, however, wasn't understood until the 1980s, when it was determined that the only reliable indicator of a Se deficit was a favorable reaction to Se therapy. Earlier, it was claimed that the usual clinical measures of plasma and urine levels did not produce sufficient findings [253]. Ashton et al. [254] and Behne et al. [255] have reviewed Se status and measures more recently.

In Lewis's work [256], a few Se levels with regard to Se status are provided. For instance, Se insufficiency manifests at intakes of 19 g/day, while Se toxicity manifests at intakes of >900 g/day. Intake averages 75 g/day in the UK versus 93 g/day for females in the USA. Contrastingly to male athletes, 66% of female athletes were reported to have Se intakes that were significantly below the French RDA.

Reduced immunological function, cardiomyopathy, skeletal muscle myopathy, osteoarthropathy, certain malignancies, and viral illness can result from selenium insufficiency at intake levels of less than 19 g/day [250]. In patients with low plasma selenium concentrations (1.2 mol/L), selenium supplementation enhances T-cell-mediated immunological responses to an oral vaccine and has been linked to faster poliovirus clearance and fewer viral mutations [257]. Recent research has demonstrated that dietary selenium deprivation can elevate inflammatory cytokines in pig brains via activating the iNOS/NF-kB pathway [258]. Heat shock proteins were revealed to be the mediators of this inflammation.

#### 3.5.3. Selenium and Infectious Diseases

Selenium plays a crucial role in cell-mediated immunity. As a result, it is anticipated that selenium deficiency would increase susceptibility to bacterial and viral infections and the resulting mortality [259].

*(1) Viral Infections*. ROS are produced in greater quantities during viral infections, which inhibit the manufacture of antioxidant enzymes in the infected cell [260]. Deficiencies in macro- and micronutrients, particularly selenium, are frequently linked to viral infections. Patients suffering from viral infections frequently have selenium deficiencies. For instance, *coxsackievirus* is an enterovirus that can cause Keshan disease, which is characterized by myocarditis and *coxsackievirus*-induced cardiomyopathy. Keshan illness is characterized by gastrointestinal distress and full-blown pericarditis. Blood selenium values below 20 g/L (0.25 M) are indicative of severe selenium insufficiency in infected patients [261, 262]. According to the available data, selenium supplementation could fully prevent the onset of Keshan disease by boosting viral immunity and promoting genetic changes in the viral genomic RNA, which together would diminish virulence and cardiac pathology. When a noncar diovirulent strain of *coxsackievirus B* (*CVB3/0*) was introduced into animal trials, only selenium-deficient mice showed signs of heart damage, but mice fed diets that were adequate in selenium (0.2 ppm of selenite) did not exhibit any signs of heart damage. The selenium-deficient mice's hearts had a greater viral load, and their antigen-specific T-cell responses were less potent than those of their littermates who had normal levels of selenium. The benign *CVB3/0* strain caused myocarditis in mice with an abnormal selenoenzyme (GPx1) gene. Most of the nucleotide connections between viruses isolated from infected GPx1// mice were similar to those in mutant viruses from selenium-deficient mice. This shows that GPx1's interaction with selenium prevents ROS from causing mutations in the viral RNA genome [263].

Acquired immunodeficiency syndrome (AIDS), which is caused by the human immunodeficiency virus (HIV), is one of the leading causes of death worldwide, but particularly in sub-Saharan Africa. Untreated HIV infection is an RNA viral infection that can lead to immune system breakdown over time. Numerous initiatives are being taken to lessen the impact of HIV infection. Of particular importance are enhancing antiretroviral therapy (ART) and encouraging dietary interventions. Although ART cannot completely cure HIV/AIDS, it can reduce viral replication and raise CD4 numbers [264]. However, the majority of sub-Saharan African countries still have limited access to ART, and its use is linked to negative side effects including altered body fat distribution, insulin resistance, and exhaustion. Due to limited patient adherence to ART, medication resistance develops [265].

A strong immune system and a balanced diet are inextricably linked. Micronutrients, in particular, are essential for the treatment and management of HIV patients. Patients with HIV frequently experience micronutrient deficiencies, including selenium deficiencies. In comparison to patients with asymptomatic HIV and the general population, patients with AIDS also showed lower levels of selenium and reduced GPx activity. Low amounts of glutathione and GPx activity in CD4+ cells raise peroxide levels, which then trigger apoptosis and kill HIV-infected cells. This suggests that there is a positive correlation between blood selenium levels and both illness severity and mortality risk. In addition, it is understood that nuclear factor kappa B (NF-B) controls the redox-controlled signal transduction system by which HIV-1 expression is regulated. In dormantly infected T cells, selenium administration can boost GPx activity, shielding them against hydrogen peroxide and lowering NF-kB activation in selenium-supplemented cells [260].

Meanwhile, greater discharge of HIV-infected cells in the vaginal tract is linked to a marginal selenium deficit in HIV patients [261]. Evidence suggests that high selenium consumption combined with other micronutrients such as vitamin combinations (B vitamins, vitamins C and E) could greatly delay the death of CD4+ cells and the beginning of AIDS, as well as the risk of comorbidities, even though results are still inconsistent. The severity of these individuals' deficiencies will determine how effective selenium treatment is. In addition, when combined with other micronutrients, selenium supplementation was more successful at enhancing patients' selenium levels as compared to selenium treatment alone. The form of selenium supplementation (selenite or selenomethionine) and the stage of HIV infection are other variables that impact how effective selenium supplementation is.

*(2) Bacterial Infections*. On whether selenium protects the body against bacterial infections, there is scant proof. Nevertheless, giving patients with *Mycobacterium* TB selenium compounds in addition to multivitamins has significantly improved their nutritional status and helped them gain weight [266]. According to studies, nutritional problems in tuberculosis patients, such as macronutrient and micronutrient deficits, malabsorption, and higher metabolic demands, enhance the severity of the illness and prolong the course of treatment [267]. Furthermore, research on animals shows that selenium levels affect the immunological response to bacterial infection. In sheep with foot rot caused by selenium deficiency, both the innate and humoral immune responses were compromised. Although it was unable to stop foot rot, selenium administration helped restore immunological function [268].

#### 3.5.4. Selenium and Cancer

The association between high selenium levels and reduced risk of cancer has been further supported by meta-analysis. Selenium exerts its influence on cancer through its impact on cell cycles, apoptosis, DNA damage and repair, cell adhesion and migration, angiogenesis, and immunology. The appropriate dosage and chemical form of selenium supplementation for cancer therapy should be determined through clinical studies [269].

By contrast, many parts of the world have poor dietary intakes of selenium. If selenium supplementation were incorporated into public health initiatives, it is anticipated that the risk of cancer and related morbidity and mortality would be greatly reduced. This is because selenium has antitumorigenic effects. In addition, selenium can sequester other elements found in food, drink, and workplace environments. A powerful detoxifying mechanism is selenium's ability to sequester these elements. Studies on animals show a link between cancer risk and long-term exposure to the aforementioned heavy metals. For instance, cadmium has been linked to a higher risk of developing prostate cancer; cadmium, chromium, and zinc to a higher risk of developing breast cancer; and cadmium, arsenic, chromium, antimony, cobalt, and lanthanum to a higher risk of developing bronchial cancer. All these components are interconnected with selenium. Thus, selenium remains a potential candidate biomarker for cancer [270].

In addition, TrxR, a newly identified selenocysteine and one of the well-documented selenoproteins in cancer, is crucial to the prevention, therapy, and diagnosis of cancer. TrxR, which is produced by preneoplastic and tumor cells, can accelerate the growth of tumors and the emergence of cancer's resistant phenotype.

#### 3.5.5. Nutritional Aspects of Selenium

The foods we eat naturally contain selenium. The following food items contain selenium: oats, wheat, brown rice, sunflower seeds, and mushrooms. Se is, however, particularly deficient in dairy, fruit, and vegetable products [103]. Brazil nuts, seafood like shrimp and oily fish, beef, and meat products are all in great supply. The amount of selenium in the diet varies regionally. Inorganic selenium found in soils is changed by plants into organic selenium. The most frequent causes of selenium insufficiency are low food intakes and soils with low selenium contents [259]. In addition, selenium supplements come in a variety of forms, including capsules, pills, powders, syrups, beverages, and energy bars [271]. Although selenium has several benefits, the body only needs a small quantity of it; therefore, consuming too much of it can be hazardous. Meanwhile, excessive use of dietary supplements may result in serious negative effects. For instance, some individuals may interact with specific medications, resulting in a variety of negative outcomes. As a result, it is advisable to take vitamins as prescribed by a doctor.

## 4. Discussion

The involvement of specific vitamins and minerals in immune function has lately been the subject of several reviews [272–281], hence Table 6 merely provides a summary of their key findings.

The scientific literature effectively emphasizes the significance of the mentioned minerals in supporting a healthy immune system. Although mineral deficiencies are rare, there are specific vulnerable populations that require special attention to ensure sufficient intake. A well-balanced diet serves as an excellent foundation in meeting these needs. In rare cases of deficiency, supplementation may be necessary; however, it is important to note that excessive consumption of certain mineral supplements can have adverse effects on the immune system. Therefore, any form of therapeutic nutrient supplementation should always be approved by a medical professional and utilized at recommended concentrations.

## 5. Conclusions

Altered zinc homeostasis, as mentioned earlier, plays a role in modulating both innate and adaptive immune responses. Deficiency in zinc can have profound effects on cellular and systemic levels, leading to increased susceptibility to infections and autoimmune diseases. Fortunately, proper zinc supplementation has been shown in numerous clinical trials to reverse the negative outcomes of zinc deficiency and restore impaired immune functions. Zinc also exhibits strong anti-inflammatory and immunomodulatory properties, making it an excellent therapeutic agent. Furthermore, ongoing research in copper biology aims to elucidate the diverse roles of this essential nutrient in various human pathologies, including neurodegenerative disorders, connective tissue disorders, cardiovascular diseases, and conditions involving disruptions in lipid metabolism. In recent years, significant progress has been made in understanding the molecular mechanisms governing microbial and mammalian iron metabolism, as well as the interplay between iron homeostasis, immunity, and disease tolerance. While numerous pieces of the biomedical puzzle have been identified, we still lack a comprehensive overview of the entire emerging picture. An important strategy for combating selenium deficiency worldwide, especially in sub-Saharan Africa, is to increase the consumption of selenium-rich foods. However, the effectiveness of this strategy is limited to regions where local food sources are naturally abundant in selenium. Manganese participates in the production of antioxidants and is essential for the proper functioning of enzymes involved in immune responses. Deficiencies in these minerals can lead to impaired immune function, increased susceptibility to infections, and altered immune responses. A healthy and balanced diet that includes a variety of nutrient-rich foods is crucial for ensuring an adequate intake of these essential minerals. In certain cases, targeted supplementation may be necessary to address mineral deficiencies, but it should be done under the guidance of a healthcare professional. However, it is important to note that excessive intake of certain mineral supplements can have adverse effects on the immune system, emphasizing the need for moderation and proper dosage. Further research is needed to better understand the intricate interactions between minerals and the immune system, as well as their potential therapeutic applications in immune-related disorders. Overall, maintaining optimal mineral status through a balanced diet and appropriate supplementation when necessary is essential for supporting a healthy and robust immune system.

## Figures and Tables

**Table 1:** General physiological functions of magnesium.

General physiological functions of magnesium [7–9]	Plays a significant role in intracellular signaling, membrane function, and enzyme activation
	Serves as a significant cofactor for numerous enzymes
	It contributes to the production of enzymes and hormones as well as the synthesis and replication of RNA and DNA
	Has a major role in oxidative phosphorylation, muscular contraction, and other metabolic processes
	Maintains the integrity of membrane components and their potential
	Through the control of iron transporters, it regulates ion transmembrane mobility

**Table 2:** General physiological functions of zinc.

General physiological functions of zinc [30–36]	Is involved in the functioning of over 300 enzymes, either as a coenzyme or a regulator, playing fundamental roles in various biochemical processes
	Participates in important functions such as DNA synthesis, protein synthesis, cell division, and gene expression
	Is involved in antioxidant defense mechanisms, immune system regulation, wound healing, and growth and development
	Participates in the production of both DNA and RNA, as well as proteins, and is a part of transcription factors
	Functions as an antioxidant and affects the structure of multiprotein complexes, including the T-cell receptor, as well as the stability of biological membranes
	Controls how hormones and their receptors are made
	Plays a key role in preserving immunological homeostasis, having an impact on the ability of cells in the innate and adaptive immune systems to function
	Regulates the synthesis of cytokines, complement system activity, and antibody formation

**Table 3:** Consequences of Zn deficiency.

Consequences of Zn deficiency	Enhanced oxidative stress, which occurs due to an imbalance between the production of reactive oxygen species and the antioxidant defense system
	Systemic inflammatory responses, due to an imbalance in the regulation of proinflammatory and anti-inflammatory processes, resulting in an exaggerated and prolonged inflammatory response
	Dysregulation of adaptive immune activation, potentially affecting the development and function of specialized T and B immune cells

**Table 4:** Examples of different food groups and their zinc content [97].

Food (preparation method and serving size)	Zinc (mg) per serving
Oysters, cooked, breaded, and fried (85 g)	74.0
Beef chuck roast, braised (85 g)	7.0
Crab, Alaska king, cooked (85 g)	6.5
Beef patty, broiled (85 g)	5.3
Breakfast cereal, fortified with 25% of the DV for zinc (18.75 g)	3.8
Lobster, cooked (85 g)	3.4
Pork chop, loin, cooked (85 g)	2.9
Baked beans, canned, plain, or vegetarian (12.5 g)	2.9
Chicken, dark meat, cooked (85 g)	2.4
Yogurt, fruit, low fat (227 g)	1.7
Cashews, dry roasted (28 g)	1.6
Chickpeas, cooked (12.5 g)	1.3
Cheese, Swiss (28 g)	1.2
Oatmeal, instant, plain, prepared with water (28 g)	1.1
Milk, low-fat, or nonfat (240 ml)	1.0
Almonds, dry roasted (28 g)	0.9
Kidney beans, cooked (12.5 g)	0.9
Chicken breast, roasted, skin removed, ½ breast	0.9
Cheese, cheddar, or mozzarella (28 g)	0.9
Peas, green, frozen, cooked (12.5 g)	0.5

**Table 5:** General physiological function of selenium.

General physiological functions of selenium	It is necessary for neutrophils, macrophages, NK cells, and T lymphocytes to operate properly
	A higher intake of selenium is associated with a lower risk of developing cancer and may help reduce inflammation and oxidative stress
	It works to increase HIV pathogenicity, which prevents the virus from developing into AIDS
	It may lower the chance of miscarriage and is necessary for sperm motility
	A lower level of selenium has been linked to an increased risk of cardiovascular disease and depressive disorders

**Table 6:** Minerals: functions, main roles in the immune system, consequences of deficiency.

Mineral	Functions [279, 280]	Main roles in the immune system [272–278]	Consequences of deficiency [272–278]
Selenium	The main forms of selenium found in animal tissues are selenomethionine and selenocysteine Is primarily associated with so-called selenoproteins, such as selenium-dependent glutathione peroxidases, to carry out its functions Protection from oxidative stress, control of thyroid hormone production, reduction and oxidation of vitamin C and other chemicals, and regulation of thyroid hormone activity	Influences both innate and acquired immunity; crucial for optimal immune response Glutathione peroxidases are required for redox control and antioxidant function by eliminating an excess of potentially harmful radicals created during oxidative stress Supplementation slows the spread of the HIV-1 viral load and raises CD4 levels	Phagocytosis of neutrophils may be compromised. Aspects of cell-mediated immunity and immunoglobulin titers are decreased by deficiency Supplementation counteracts these effects May be a factor in some malignancies, viral illnesses, lowered immune function, and generally increased vulnerability to infections Because of a lack of nutrients, viruses mutate into more dangerous varieties

Zinc	An essential nutrient with profound impacts on the immune system, collagen formation, and antioxidant defenses, as well as importance in cellular growth and differentiation More than 300 enzymes and proteins, as well as a component of 1,000 transcription factors, including DNA-binding proteins with zinc fingers, depend on it for the catalytic function necessary for biological activity A factor in the control of gene expression	Acts on cellular and humoral immunity, preserving the integrity of the skin and mucous membranes Protection of cells from the harm caused by reactive oxygen and nitrogen species created during immunological activation	Deficiency reduces neutrophil and macrophage phagocytosis, NK cell activity, the production of oxidative bursts, and complement activity Thymus involution, reduced lymphocyte growth, generation of T cells, DTH skin reactions, and antibody response Heightened infection risk, especially for young people and the elderly Atrophy of lymphoid organs Adverse effects on bone marrow

Iron	Essential part of myoglobin, which transports and stores oxygen in the muscle and releases it as needed during contraction, and hemoglobin for carrying oxygen Facilitates electron transport in the respiratory chain, which is crucial for ATP generation Red blood cell production and function depend on it Constituent of many enzymes Reduces the risk of macrocytic hypochromic anemia	Keeps the skin and mucous membranes healthy by affecting cellular and humoral immunity Reactive oxygen and nitrogen species that injure cells when the immune system is engaged are protected from in cells Important for immune cell function necessary for T-cell responses (DNA synthesis, ribonucleotide reductase), formation of reactive oxygen species, and essential for cell development and proliferation Regulating the synthesis and function of cytokines Involved in the production of extremely toxic hydroxyl radicals, which neutrophils use to destroy germs	Reduced levels of circulating monocytes, lymphocytes, CD4 cells, the CD4/8 ratio, cytotoxic T lymphocyte and NK cell activity, and neutrophil respiratory burst Supplementation can reverse such alterations Increased risk of infection Lymphoid organ atrophy The immune system suffers negative functional effects when cellular iron homeostasis changes due to either a shortfall or an overload

Copper	Involved in the production of proteins, the neurological system, bone health, and the metabolism of iron Aerobic oxidation's crucial cofactor for cytochrome C oxidase Essential for antioxidant action and generating energy	Cu/Zn-superoxide dismutase, a crucial enzyme in the fight against reactive oxygen species, contains this component Maintains the equilibrium of intracellular antioxidants, indicating a key function in the inflammatory response Changes in homeostasis play a significant influence in the innate immune response (macrophages, neutrophils, and monocytes), which is a major component of respiratory burst	Neutropenia is characterized by abnormally low neutrophil counts and reduced mononuclear cell growth Infection severity and susceptibility to infections have both increased Deficiency is associated with a rise in animal virulence Immune response is adversely affected by both shortage and surplus

## Data Availability

No data is available for this study.
